# A Halt in Life's March

**Published:** 1887-12-24

**Authors:** 


### The Hospital

A Weekly Institutional Journal of

**Science, Medicine, and Philanthropy.**

*For Hospitals, Asylums, Medical Practitioners, Students, Nurses, Families, Parishes, Congregations, and the Charitable Public.*

Vol. III. No. 65.] [December 24, 1887.

### A Halt in Life's March.

There are many and various opinions in the world as to the value and permanency of the Christian faith, but most people in this country are agreed that Christmastide is a wholesome and pleasant season. A halt is called in the march of business, arms are grounded in the conflict of life, and a brief breathing time is given to us all. "It is better to go to the house of mourning than to go to the house of feasting," said Solomon. Nevertheless, feasting, too, has its proper season, and in its season it is most welcome and right. "It is a poor heart that never rejoices," says the North country proverb, a sentiment in which we entirely concur. If the whole world could come together into one huge banqueting hall, and for one brief day forget its toils and strifes in a grand feast of human brotherhood, a new epoch in civilisation might be dated from that period. One of the fundamental ideas of the faith whose origin we celebrate on Christmas Day, is this of the common brotherhood. In principle few of us doubt it, and at Christmas time there is, by general consent, an united effort to put the principle into practice. The poor look to the rich to help them to celebrate the season worthily, and the rich most joyfully respond to the appeal with kind words and generous deeds.

The world is not such a very bad place after all; and he who has a wholesome and friendly spirit generally meets among all ranks of his fellow men some who will reciprocate his warm and friendly impulses. The human husk is often tough and unlovely, and the shell within the husk is sometimes exceedingly difficult to crack; but in most human beings there is a kernel of sterling worth, even though in many it may be shrivelled and dry. It is well sometimes to insist upon this fact, otherwise the faith in human goodness is in danger of being extinguished. We are never so ready to believe in the warmth of other people's hearts as when our own are alive with a generous flame. At Christmas time all the embers of human kindness are raked together, and the Yule log is put on, so that some which were in danger of expiring are rekindled by the general heat, and blaze up afresh for another year.

Few things are more instructive than the changed estimate of certain persons which takes place under favourable circumstances. Nobody can say that Mr. Labouchere is regarded as a saint by any considerable number of the general public. Probably he himself would be the first to smile at any suggestion of such a distinction. Yet by virtue of his *Toy Fund* Mr. Labouchere at Christmas time becomes one of the heroes of the hour. The Toy Fund idea at the outset was so excellent; it contained so much of that "nature" of which even a "touch makes the whole world kin," that every one feels the man must have some "soul of good" in him whose heart could be the birthplace of such a thought. But the idea has not remained an idea; it has germinated and sprung up, and borne fruit; and now, like the grain of mustard-seed in the parable, it has become a large tree spreading its influence over all the land. We cannot always admire Mr. Labouchere's public utterances, or the lines which he lays down for himself; but we can and do admire the toy-world of which he has been the creator; and we doubt not that many a gentle child would speak trustful words to Mr. Labouchere who would turn away from more religious and more austere men. No one in all the world was ever so ready to give full recognition to a good deed as was He whose birth we celebrate on Christmas Day. The deeds done for the children's sake are not forgotten by Him. "Inas much as ye have done it unto one of the least of these My brethren, ye have done it unto Me." Every hospital child is waiting with eager expectation for the dawn of the Christmas Day, which brings Santa Claus with his welcome load of toys. It is a proud distinction to be Santa Claus to ten thousand hospital children. We may all envy Mr. Labouchere his feelings as the hour of distribution approaches. Probably no event in his life has given him so much sincere pleasure. Such is the magic of good deeds to those who perform them.

Mr. Labouchere would be the last to wish to monopolise all the honour of rendering good service to hospitals at Christmas time. There are other and more substantial things than toys needed; and many whose names will never be known are found ready to supply them. For after all that can be said it is the steady supporters of hospitals all the year round who bear the brunt of the battle, and for whom our warmest commendations must be reserved. These are they who from the high principles of obedience to Him who was born on Christmas Day devote year by year a definite portion of their substance to works of charity. Hospitals, though not exclusively, are pre-eminently Christian institutions. They were in existence as far back as 1134 B.C., and among the Buddhists were by no means uncommon. But it is to Christianity that they owe their permanent establishment on the principles of voluntary charity, as well as their universal diffusion; and it is on the sentiment of brotherhood, and the peculiar ideas of duty which spring from the Christian faith, that they depend for their constant maintenance and development. In saying this we cast no reflection upon other forms of religion. We are well aware that our Jewish fellow-citizens are among the most generous and constant supporters which hospitals have ever had. Still, in this country the prevalent faith is the Christian, and it is to Christians especially that we turn when any appeal is to be made on behalf of the whole poor and suffering class.

If we could have our way, we should persuade the public on two or three definite points. One would be that they should take a little more trouble and give a little more thought to their charitable work. They should find out by careful inquiry those institutions which possess the two characteristics of "need" and "merit." It is no charity to give to those who do not need; and it is no virtue to give to those who do not deserve. At this special season, too, we should try to induce those who can well afford it to seek out some particular institution which is in special need; as, for example, any hospital which may be erecting new buildings, or any in an exceptionally poor neighbourhood, or which may be burdened with debt. Those who can spare large sums might render infinite service by concentrating their energies on one definite and thoroughly deserving hospital.

Almost all people, even those who have but little to spare, turn kindly towards the poor and the sick at Christmas time. Gentle thoughts are to be cherished, and to be nourished until they grow to deeds. Such thoughts entirely fail of their value if they end with mere vague wishes. They bless neither the thinker nor those who are thought about. Both the hospitals and their inmates expect and hope for a liberal share of the kindness and generosity of the season. We would fain feel assured that every hospital, even the least known, and every patient, the most poor and insignificant, may be specially remembered by some good and friendly soul. Whatever be a man's religion or no-religion, he must be less than human if he does not feel that kindthoughts and good deeds done to the sick, the suffering, and the poor, are among the highest privileges which belong to him as a sensitive and reasonable being. "Blessed is he that considereth the poor."

We do not wish to claim for hospitals an exclusive regard at this season of festivity. Although they are in a sense our special province, or at least one of our special departments of public work, yet our active interest extends to all the poor and needy, and to every agency used for their relief and restoration to work or health. It is exceedingly to be desired that all who are even moderately prosperous should bring their thoughts and the generous feelings of their hearts to bear upon the problem of the sorrows and distresses of the poor at this season of unusually stern pressure. It may be taken as absolutely certain that very many men and women of excellent character, and a vast number of helpless and quite irresponsible children, are suffering all the dread extremities of slow starvation by cold and hunger. Looked at from any standpoint, it must be terrible to those poor creatures to see every town and city filled with rejoicing abundance whilst they themselves feel the pangs of hunger, and have not so much as even bread to eat, clothing to wear, or fire to warm. We would not mar anybody's Christmas pleasure by reproaches, but we would take upon ourselves the needs and sorrows of the poor, and plead for immediate and ungrudging help.

